# Can’t Dissolve Me Now: A COVID-19 Provoked Venous Thromboembolism Breaks Through Apixaban: Case Report

**DOI:** 10.5811/cpcem.2021.3.50505

**Published:** 2021-04-28

**Authors:** Alexander W. Arena, Ahmad Hussein, Ellen J. Kurkowski, Miriam L. Kulkarni

**Affiliations:** St. John’s Riverside Hospital, Department of Emergency Medicine, Yonkers, New York

**Keywords:** Case report, COVID-19, endotheliopathy, venous thromboembolism, apixaban

## Abstract

**Introduction:**

Coronavirus disease 2019 (COVID-19) is a multisystem process with a growing evidence of its endotheliopathy effects, with subsequent hypercoagulability states.

**Case Report:**

WWe present an emergency department case of a COVID-19-provoked deep venous thrombosis and pulmonary embolism without a history of venous thromboembolism (VTE), with extension of the VTE despite adherence to apixaban.

**Conclusion:**

This case demonstrates the importance of further research and protocols for optimal dosage and treatment to prevent worsening VTE in COVID-19 patients.

## INTRODUCTION

By the end of 2019, a novel coronavirus was identified as the pathogen causing a collection of respiratory cases to appear in Wuhan, China. In early 2020 the World Health Organization designated this new disease as coronavirus disease 2019 (COVID-19).^1^ Severe acute respiratory syndrome coronavirus 2 (SARS-CoV-2), the virus that causes COVID-19, is a non-segmented, positive-sense ribonucleic acid virus that is part of the family of coronaviruses that includes SARS and Middle East respiratory syndrome due to its high homology.^2^ It is known that SARS-CoV-2 binds via the angiotensin-converting enzyme 2 receptor that is located along intestinal epithelium, vascular endothelium, and type two alveolar cells.^3^

Initially, it was believed that COVID-19, due to its injurious mechanism to the vascular endothelium, caused acute respiratory distress syndrome (ARDS).^4^ However, studies have shown the hypercoagulability state associated with COVID-19. This is evidenced by the endothelial injury from viral particles inside endothelial cells on autopsy, increased angiogenesis, and elevated circulating prothrombotic factors such as D-dimer, fibrinogen, and von Willebrand factor.^5^ At the time of writing, there were over 19 million cases of coronavirus throughout the world, with cases in the United States just over three million, and still with a linear rise.^6^

We present a case of a 59-year-old female with a recent history of an active COVID-19 infection who was diagnosed with an acute extension of deep vein thrombosis (DVT) despite a month of anticoagulation therapy for her previously diagnosed pulmonary embolism (PE) and DVT.

## CASE REPORT

**Image 1:**
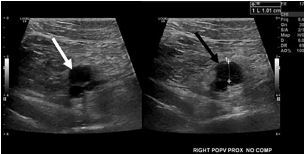
Ultrasound image of the popliteal vein on the right side without compression (white arrow) and with compression (black arrow). The popliteal vein was uncompressible, consistent with a venous thrombosis.

**Image 2:**
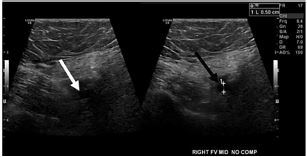
Ultrasound image of the femoral vein on the right side without compression (white arrow) and with compression (black arrow). The femoral vein was uncompressible, consistent with a venous thrombosis.

A 59-year-old female presented to the emergency department (ED) with a chief complaint of right lower extremity pain and swelling. Vital signs at triage were temperature (oral) 98.2° Fahrenheit; heart rate 83 beats per minute; respiratory rate 17 breaths per minute; pulse oximetry 99% on room air; and blood pressure of 135/78 millimeters of mercury (mm Hg). The patient had a hospital admission 40 days prior to presentation for PE and right popliteal DVT with a positive nasopharyngeal COVID-19 test. She denied a familial history of hypercoagulable states or a personal history of thromboembolic disease prior to her COVID-19 infection. In the ED, she underwent an ultrasound of the right lower extremity. The ultrasound showed the previously seen right popliteal DVT (Image 1), as well as interval development of right mid-femoral DVT (Image 2).

Repeat COVID-19 nasopharyngeal swab testing was negative. Per the patient, when she was discharged previously she was unable to follow up with a hematologist/oncologist due to COVID-19-related lockdown restrictions but maintained compliance with her apixaban at five milligrams (mg) twice per day. Her international normalized ratio (INR) on presentation was 1.36 (reference range: 0.83–1.09) and her D-dimer was 2054 nanograms per milliliter (ng/mL) (0–500 ng/mL). She was admitted for anti-coagulation and started on low molecular weight heparin (LMWH) at 1 mg per kilogram. Throughout her hospital course, she was placed on warfarin with INR values ranging from 0.98 to 4.14. An echocardiogram was performed that showed a normal size, thickness, and function of the left ventricle. In addition, the right ventricle was normal in size and function. The patient was discharged with 12 mg of warfarin daily with hematology follow-up.

**CPC-EM Capsule**

What do we already know about this clinical entity?

*COVID-19 (coronavirus disease 2019) induced endotheliopathy is known to exist with patients afflicted with the virus. As such, they develop deep venous thrombosis and pulmonary embolism.*

What makes this presentation of disease reportable?

*This is one of the few known cases of extended venous thromboembolism during COVID-19 despite direct oral anticoagulation.*

What is the major learning point?

*COVID-19 induced endotheliopathy treatment cannot be universal because of potential risk of recurrent venous thromboembolism.*

How might this improve emergency medicine practice?

*This will improve practice by helping physicians consider using anticoagulation in COVID-19 patients and consider venous thromboembolism as a differential diagnosis.*

**Image 3:**
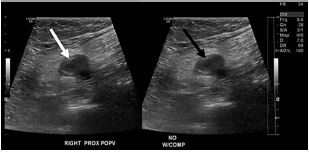
Ultrasound image from the patient’s previous admission showing the popliteal vein on the right side without compression (white arrow) and with compression (black arrow). The popliteal vein at the time of this presentation was uncompressible, consistent with a venous thrombosis.

The patient had been admitted approximately 40 days prior for submassive bilateral central PE with a right popliteal DVT (Image 3). A detailed report of her previous admission is warranted given the patient’s previous DVT and PE findings with a positive COVID-19 nasopharyngeal swab test.

On ED arrival during the first admission, vital signs at triage were heart rate 120 beats per minute; blood pressure of 150/109 mm Hg; respiratory rate of 22 breaths per minute; and pulse oximetry of 89% on room air. Laboratory studies revealed a 0.89 ng/mL troponin I (0.00–0.05 ng/mL) with the additional following labs: D-dimer 5397 ng/mL and INR of 1.0. The chest computed tomography angiography showed acute bilateral central PE with probable right heart strain with multiple small bilateral peripheral ground glass infiltrates suggestive of COVID-19 pneumonitis. The patient was given at the time a heparin infusion with a bolus based on actual body weight and protocols.

She was admitted to the intensive care unit (ICU) and underwent urgent thrombolysis with thrombectomy. During the procedure, one mg per hour of alteplase was catheter directed while mechanical thrombectomy was performed. The total amount of alteplase received was 24 mg. Repeat angiography approximately 24 hours later showed significant improvement in blood flow to the right and left main as well as segmental and subsegmental pulmonary arteries with significant decrease in central clot burden but with residual central filling defects. An echocardiogram was performed during her admission. However, due to all images being suboptimal in quality, the study was technically limited with “probable normal LV systolic function…right ventricle is not well visualized.” During her hospital course, she was placed on 10 mg of apixaban twice a day for one week and transitioned to 5 mg twice a day with which she was discharged on the same schedule. The patient underwent rehabilitation, which included daily exercising. She stated she was compliant with her apixaban medication at 5 mg twice a day.

## DISCUSSION

Since the initial stages of COVID-19, our understanding of the disease process has expanded to encompass multiple organ systems. While initially COVID-19 was associated with respiratory failure meeting the Berlin Criteria for ARDS, there has been a disseverment between pulmonary function and gas exchange at the alveolar level. There have been cases where patients with refractory respiratory failure while on mechanical ventilation with shock improved quantitatively after administration of alteplase.^7^ Postmortem examination of 21 individuals with COVID-19 found microthrombi within the alveolar capillaries of 45% of the individuals.^8^ The evidence of dysregulation of the coagulation cascade became apparent when the incidence of VTE was highest within the ICU, sometimes accounting for 69% of cases.^9^

Direct oral anticoagulants (DOAC) can be used prophylactically to reduce the incidence of VTE in patients hospitalized for acute infectious lung diseases.^10^ The use of DOACs can stabilize the clot to prevent further embolization from the vessel. However, a persistence of VTE despite anticoagulation with extension of VTE presents as a rare event. A meta-analysis performed to evaluate the rates of treatment failures in those patients treated with oral anticoagulants showed that one of the most common manifestations of treatment failure was the development of VTE with more than half of the patients transitioning to a vitamin K antagonist after DOAC failure.^11^

There have been numerous publications in regard to endotheliopathy in COVID-19 patients. Recently, a cross-sectional study found that patients in the ICU had significant elevation of markers of endothelial cell and platelet activation compared to non-ICU patients.^12^ The patients were noted to have von Willebrand factor antigen concentrations markedly elevated with mortality correlation.^12^ In addition, the endotheliopathy process is unique in comparison to other viral infections, namely influenza A virus subtype H1N1 (H1N1). In postmortem studies, alveolar capillary microthrombi were nine times as prevalent in patients with COVID-19 as compared to patients with H1N1.^13^ Furthermore, these studies found evidence of a higher density of intussusceptive angiogenesis as compared to H1N1 patients.^13^

While COVID-19 and H1N1 shared patterns of diffuse alveolar damage, COVID-19 was distinctive in that the thrombi were more diffuse throughout the pulmonary vasculature.^13^ Due to the uncertainty of what the proper dosage is for prophylaxis for VTE in patients with COVID-19, there has been increasing debate that current dosages may not be sufficient to provide adequate prophylaxis.^14^ In a randomized, double-blinded study of VTE patients treated with apixaban compared to placebo, recurrent VTE occurred in 8.8% of patients who received placebo compared to 1.7% who received 5 mg of apixaban.^15^ Given the low recurrence rate, it is plausible that recurrence of thromboembolic events are mediated by the COVID-19 disease process. While the evidence of COVID-19-induced hypercoagulability exists, there is limited data on how long it lasts. Thus, various institutions have developed protocols suggesting anticoagulation throughout the hospitalization with agents such as LMWH when admitted for COVID-19.

## CONCLUSION

Propagation of a known VTE in the setting of oral anticoagulation use is a rare event. This rare case outlines a patient with no prior medical history or risk factors for VTE or PE other than having COVID-19 with an extension of a VTE despite over 40 days of anticoagulation use. This case suggests further research and discussion is needed to properly evaluate the pharmaceutical anticoagulation choice when treating patients with COVID-19 and VTE.
